# Receptor-targeted liposome-peptide-siRNA nanoparticles represent an efficient delivery system for MRTF silencing in conjunctival fibrosis

**DOI:** 10.1038/srep21881

**Published:** 2016-02-24

**Authors:** Cynthia Yu-Wai-Man, Aristides D. Tagalakis, Maria D. Manunta, Stephen L. Hart, Peng T. Khaw

**Affiliations:** 1 National Institute for Health Research (NIHR) Biomedical Research Centre at Moorfields Eye Hospital NHS Foundation Trust and UCL Institute of Ophthalmology, London, United Kingdom; 2 Wolfson Centre for Gene Therapy of Childhood Disease, UCL Institute of Child Health, London, United Kingdom

## Abstract

There is increasing evidence that the Myocardin-related transcription factor/Serum response factor (MRTF/SRF) pathway plays a key role in fibroblast activation and that knocking down MRTF can lead to reduced scarring and fibrosis. Here, we have developed a receptor-targeted liposome-peptide-siRNA nanoparticle as a non-viral delivery system for MRTF-B siRNA in conjunctival fibrosis. Using 50 nM siRNA, the *MRTF-B* gene was efficiently silenced by 76% and 72% with LYR and LER nanoparticles, respectively. The silencing efficiency was low when non-targeting peptides or siRNA alone or liposome-siRNA alone were used. LYR and LER nanoparticles also showed higher silencing efficiency than PEGylated LYR-P and LER-P nanoparticles. The nanoparticles were not cytotoxic using different liposomes, targeting peptides, and 50 nM siRNA. Three-dimensional fibroblast-populated collagen matrices were also used as a functional assay to measure contraction *in vitro*, and showed that MRTF-B LYR nanoparticles completely blocked matrix contraction after a single transfection treatment. In conclusion, this is the first study to develop and show that receptor-targeted liposome-peptide-siRNA nanoparticles represent an efficient and safe non-viral siRNA delivery system that could be used to prevent fibrosis after glaucoma filtration surgery and other contractile scarring conditions in the eye.

RNA interference (RNAi) is a promising therapeutic approach as it can be used to silence the expression of harmful genes in a wide range of diseases^1^. Small interfering RNAs (siRNAs) are double-stranded RNA molecules 20–25 nucleotides long that regulate gene expression by driving a target messenger RNA to degradation, thus leading to gene silencing^2^. Delivery of naked siRNAs is however unlikely to be effective as siRNAs do not cross cell membranes and are susceptible to degradation by RNAses. Viral delivery systems like adenoviral vectors have been used to deliver shRNAs but carry the risk of oncogenicity and immunogenicity^3^,^4^. As a result, there is now increasing interest in developing non-viral methods, in particular nanoparticles, as a safe and efficient siRNA delivery system^5^,^6^.

There is also growing interest in developing potential siRNA therapeutics in the eye. Different research groups are studying siRNA-based therapeutic strategies in herpetic stromal keratitis^7^, retinoblastoma^8^, and ocular inflammation^9^. The synthetic siRNA, QPI-1007, inhibits the expression of the caspase 2 protein and is being developed as a neuroprotective treatment in non-arteritic anterior ischemic optic neuropathy and glaucoma^10^. Studies using intravitreal injections of siRNAs that inhibit vascular endothelial growth factor, namely siRNA-027 and PF-04523655, have also reached Phase I and II trials in age-related macular degeneration^11^,^12^.

Glaucoma is the leading cause of irreversible blindness and affects over 70 million people worldwide^13^. Subconjunctival fibrosis and contraction of the drainage channel created to lower intraocular pressure are the main causes of failure of glaucoma surgery, and human Tenon’s fibroblasts (HTFs) represent the major cell type contributing to the fibrotic process. Fibrosis also plays a part in either the pathogenesis or failure of treatment of virtually all the blinding diseases in the world today. There is now increasing evidence that the Myocardin-related transcription factor/Serum response factor (MRTF/SRF) pathway plays a key role in fibroblast activation and that knocking down MRTF can lead to reduced scarring and fibrosis^14^,^15^,^16^. We have also recently described how the MRTF/ SRF pathway is intricately linked to all the key regulators and pathways in ocular fibrosis^17^.

Furthermore, we have previously described the use of liposome-peptide nanoparticles for both *in vitro* and *in vivo* gene delivery in the airway epithelium^18^,^19^, cancer^20^,^21^, and vascular tissues^22^,^23^. The liposome-peptide-siRNA nanoparticles with their synergistic lipid and peptide components can effectively package the siRNAs and protect them from enzymatic cleavage, can be dissociated by heparin, and are localised in the cytoplasm following transfection^5^,^24^. We have also developed PEGylated formulations to further increase the receptor-targeted specificity and transfection efficiency in cells and to enable better biocompatibility of the nanocomplexes^25^,^26^.

In this study, we have developed receptor-targeted liposome-peptide-siRNA nanoparticles as an efficient non-viral delivery system for MRTF-B siRNAs in human Tenon’s fibroblasts to prevent post-surgical fibrosis after glaucoma filtration surgery and other fibroblast-induced contractile scarring conditions in the eye.

## Results

### Biophysical properties of the liposome-peptide-siRNA nanoparticles

**Figure 1:**
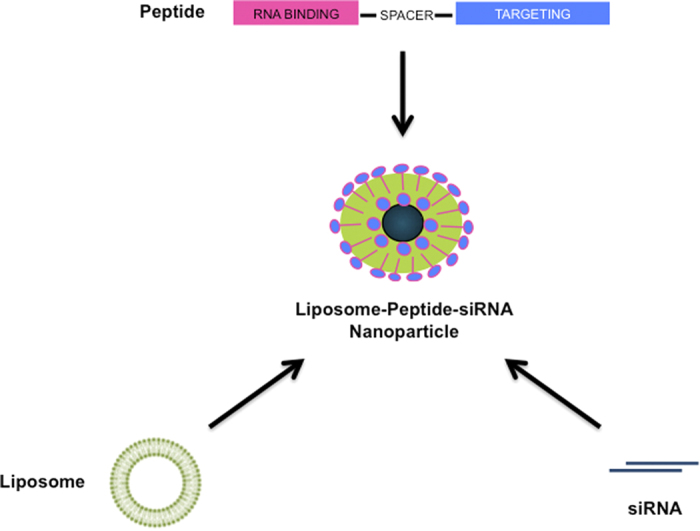
Schematic diagram of a receptor-targeted liposome-peptide-siRNA nanoparticle.

**Figure 2:**
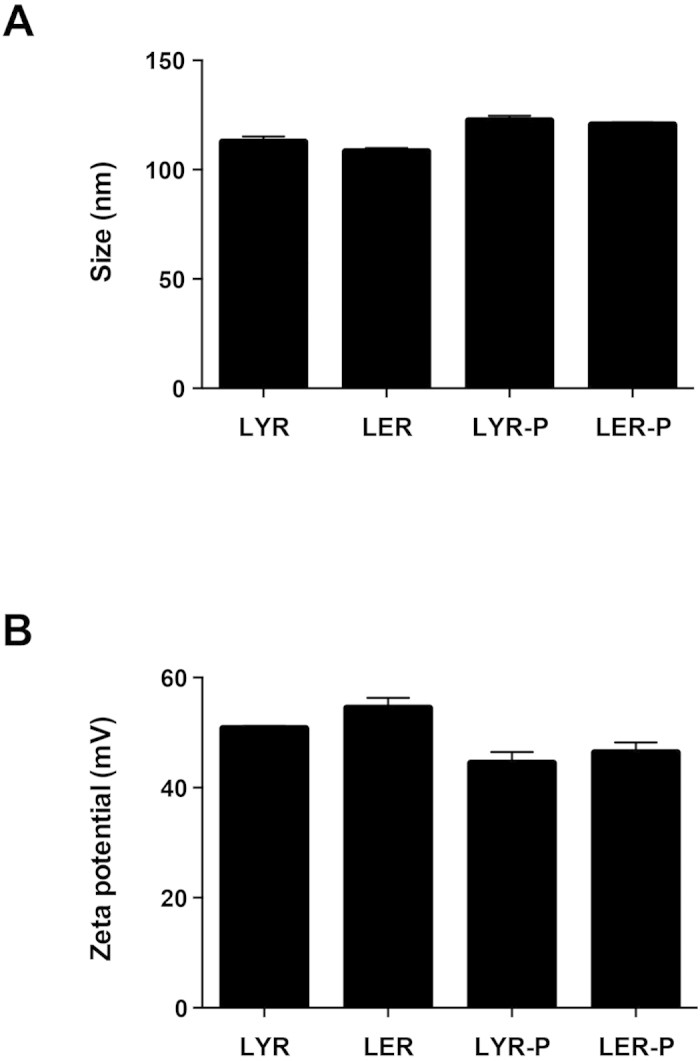
Biophysical properties of the nanoparticles with different peptide and liposome formulations. (**A**) Size in nm; **(B)** Zeta potential in mV.

**Figure 3:**
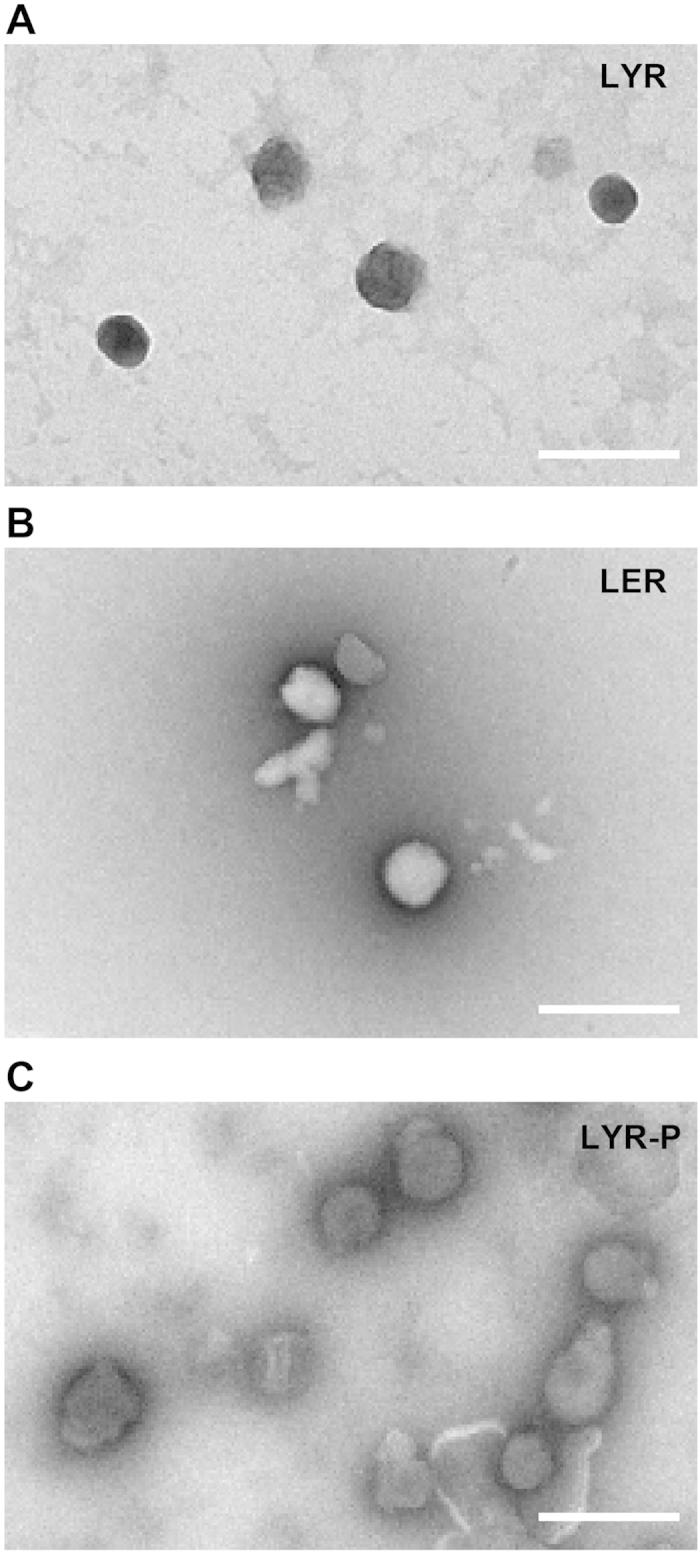
Negative staining transmission electron microscopy was used to visualise the nanoparticles. (**A**) LYR; (**B**) LER; **(C)** LYR-P. Most of the nanoparticles were spherical in morphology, Scale = 200 nm.

Figure 1 is a schematic diagram of a receptor-targeted liposome-peptide-siRNA nanoparticle prepared at a weight ratio of 1 (liposome): 4 (peptide): 1 (siRNA). All nanoparticles were strongly cationic ( + 42 to + 56 mV), with sizes around 100 nm and polydispersity indices (PDIs) less than 0.35. The LYR (non-PEGylated liposome-peptide Y-siRNA) nanoparticles measured 112.5 ± 2.6 nm (SD) and the zeta potential was + 50.7 ± 0.5 mV (SD) (Fig. 2A,B). The LER (non-PEGylated liposome-peptide ME27-siRNA) nanoparticles measured 108.2 ± 1.7 nm (SD) and the zeta potential was + 54.4 ± 1.9 mV (SD). The LYR-P (PEGylated liposome-peptide Y-siRNA) nanoparticles measured 122.4 ± 2.4 nm (SD) and the zeta potential was + 44.4 ± 2.0 mV (SD) (Fig. 2A,B). The LER-P (PEGylated liposome-peptide ME27-siRNA) nanoparticles measured 120.4 ± 1.2 nm (SD) and the zeta potential was + 46.3 ± 1.9 mV (SD). Negative staining transmission electron microscopy (TEM) was also used to visualise the nanoparticles and showed that most of the nanoparticles were spherical in morphology (Fig. 3).

### Liposome-peptide-siRNA nanoparticles demonstrate high silencing efficiency in human Tenon’s fibroblasts

**Figure 4:**
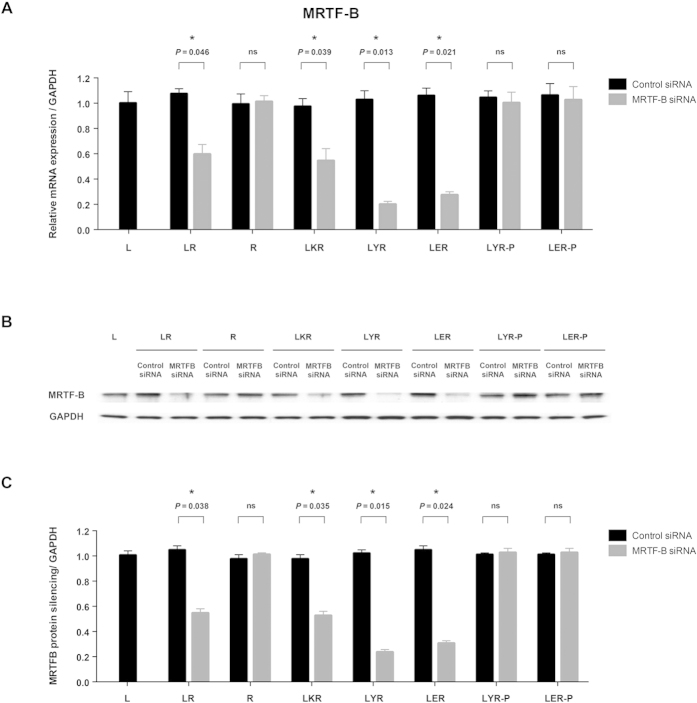
Silencing efficiency of the liposome-peptide-siRNA nanoparticles with different peptide and liposome formulations. (**A**) Using 50 nM siRNA, the *MRTF-B* gene was efficiently silenced by 76% and 72% with LYR and LER nanoparticles, respectively. LYR and LER nanoparticles showed higher silencing efficiency than PEGylated LYR-P and LER-P nanoparticles. mRNA levels were normalised relative to GAPDH and the results shown are mean ± SD for triplicate experiments; **(B**) Western blotting showed a significant decrease in the MRTF-B protein expression with LYR and LER nanoparticles; (**C**) MRTF-B protein silencing was calculated using densitometric analysis and GAPDH as loading control.

We compared the silencing efficiency of liposome-peptide-siRNA nanoparticles in human Tenon’s fibroblasts using different targeting peptides (Y, ME27) and different liposomes (non-PEGylated, PEGylated). Using 50 nM siRNA, the *MRTF-B* gene was efficiently silenced by 76% and 72% with LYR and LER nanoparticles, respectively (Fig. 4A). The expression of the MRTF-B protein was also significantly decreased after treatment with both LYR and LER nanoparticles (Fig. 4B,C). We have performed control experiments for DOTMA/DOPE/ K_16_/ siRNA (LKR), DOTMA/DOPE/ siRNA (LR), DOTMA/DOPE only (L), and siRNA only (R). The targeting formulations (LYR and LER) demonstrated better silencing efficiency than LKR, a non-targeting control, or LR, emphasising the importance of targeting in efficient gene silencing (Fig. 4A–C). There was also poor silencing efficiency when siRNA only or DOPMA/DOPE only were used, showing that delivery of naked siRNAs is not sufficient to cross the cell membrane. In addition, we found that LYR and LER nanoparticles showed higher silencing efficiency than PEGylated LYR-P and LER-P nanoparticles in human Tenon’s fibroblasts (Fig. 4A–C). The internalisation of the nanoparticles was also examined by confocal microscopy and LYR nanoparticles showed higher cellular uptake than PEGylated LYR-P nanoparticles (Supplementary Figure 1).

**Figure 5:**
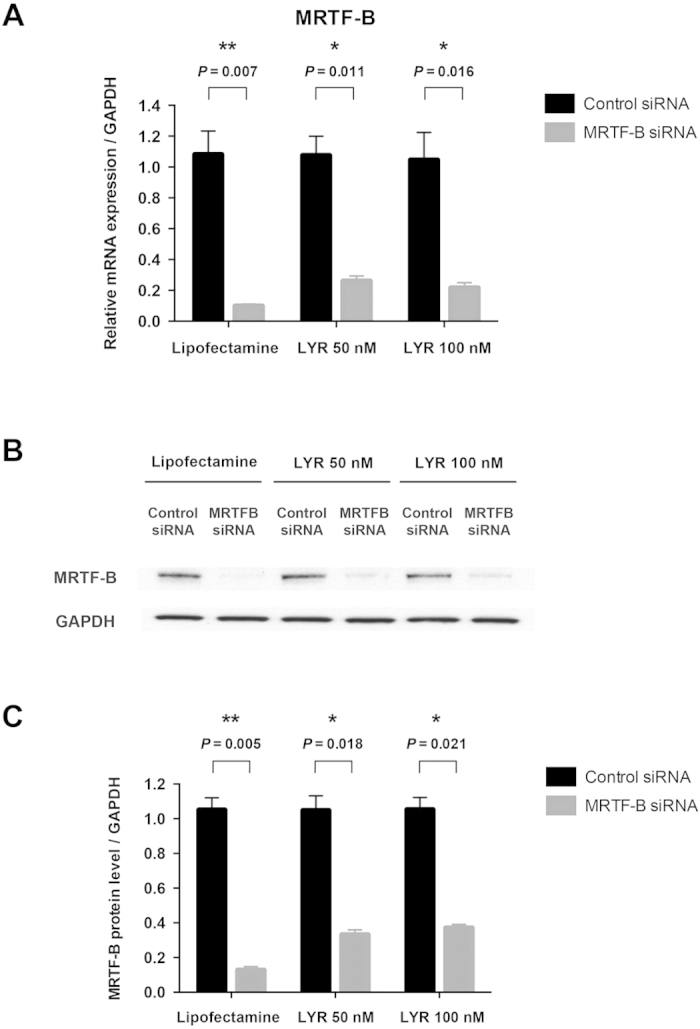
Silencing efficiency of LYR nanoparticles with different siRNA concentrations. (**A**) The expression of the *MRTF-B* gene was efficiently silenced by 76% and 80% at 50 nM and 100 nM siRNA respectively, compared to the 91% achieved using lipofectamine reagent at 50 nM siRNA; **(B**) Western blotting also showed a marked reduction in the MRTF-B protein levels after treatment with MRTF-B LYR nanoparticles at both 50 nM and 100 nM siRNA; (**C**) MRTF-B protein silencing was calculated using densitometric analysis and GAPDH as loading control.

As LYR nanoparticles showed higher transfection efficiency, we further studied LYR nanoparticles at a higher siRNA concentration of 100 nM compared to 50 nM. We found that MRTF-B LYR nanoparticles efficiently silenced the *MRTF-B* gene by 76% and 80% using 50 nM and 100 nM siRNA concentrations respectively, compared to 91% using lipofectamine reagent at 50 nM siRNA (Fig. 5A). The results were confirmed at protein level and there was a marked reduction in the MRTF-B protein expression after treatment with MRTF-B LYR nanoparticles at both 50 nM and 100 nM siRNA concentrations (Fig. 5B,C).

### Liposome-peptide-siRNA nanoparticles represent a safe delivery system in the conjunctiva

**Figure 6:**
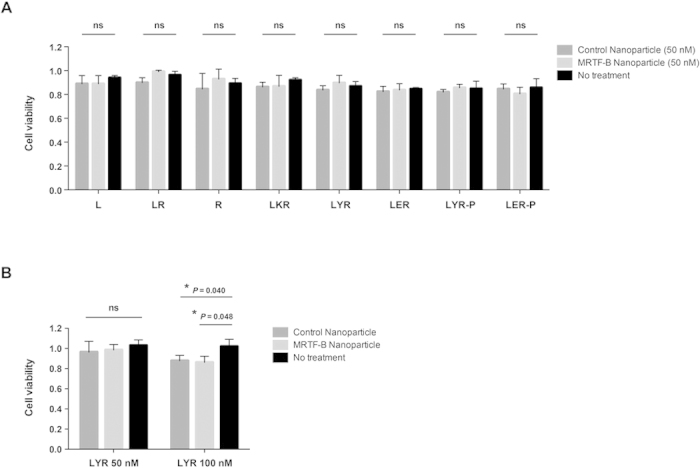
Cytotoxicity of nanoparticles. (**A**) Using 50 nM siRNA, MRTF-B nanoparticles with different targeting peptides and liposomes (LYR, LER, LYR-P, LER-P) did not significantly affect cell viability compared to control nanoparticles and untreated cells; (**B**) A decrease in cell viability was however noted with both MRTF-B and control LYR nanoparticles at 100 nM siRNA compared to untreated cells. Results shown are mean ± SD for six independent replicates.

We next compared the cytotoxicity of the liposome-peptide-siRNA nanoparticles in human Tenon’s fibroblasts using different targeting peptides (Y, ME27) and different liposomes (non-PEGylated, PEGylated). Using 50 nM siRNA, the MRTF-B LYR and LER nanoparticles were not cytotoxic compared to control nanoparticles and untreated cells (Fig. 6A). In addition, there were no statistically significant differences in cell viability when MRTF-B PEGylated LYR-P and LER-P were used compared to control nanoparticles and untreated cells (Fig. 6A).

We further compared the cytotoxicity of LYR nanoparticles in human Tenon’s fibroblasts at the higher siRNA concentration of 100 nM compared to 50 nM. Although the LYR nanoparticles were not cytotoxic at 50 nM siRNA, a decrease in cell viability was however noted with both the MRTF-B and control LYR nanoparticles at 100 nM siRNA compared to untreated cells (Fig. 6B).

### Liposome-peptide-siRNA nanoparticles prevent matrix contraction after a single transfection treatment

**Figure 7:**
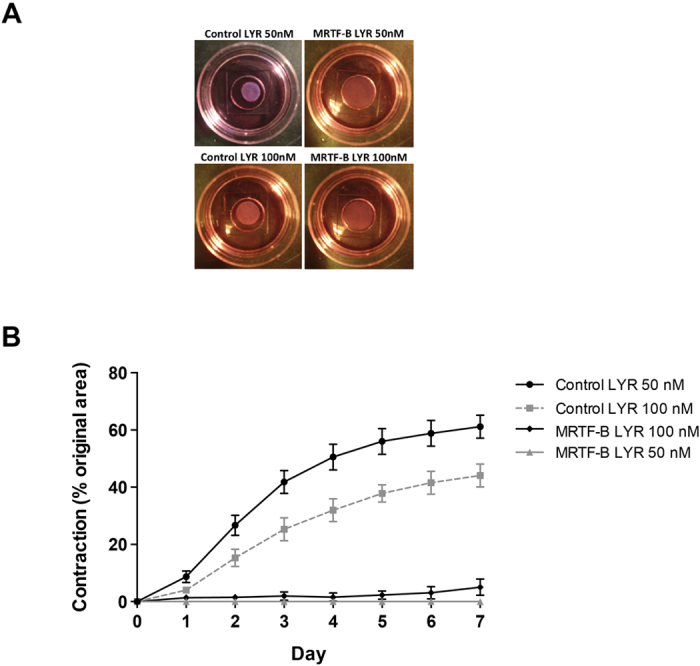
The MRTF-B LYR nanoparticles prevented collagen matrix contraction after a single transfection treatment compared to control LYR nanoparticles. (**A**) Representative gel areas at day 7 of contraction assay; (**B**) The MRTF-B LYR nanoparticles showed the maximal decrease in matrix contraction at 50 nM siRNA compared to 100 nM siRNA.

We used detached three-dimensional fibroblast-populated collagen gels as these have been shown to be a very good *in vitro* model and functional assay to study tissue contraction in the eye^27^,^28^. Figure 7A shows representative gel areas at day 7 of the contraction assay. The MRTF-B LYR nanoparticles effectively blocked collagen matrix contraction for the whole duration of the 7-day contraction assay after a single transfection treatment, compared to control nanoparticles (Fig. 7B). The MRTF-B LYR nanoparticles showed the maximal inhibition in contraction at 50 nM siRNA compared to 100 nM siRNA.

We also noted that the fibroblasts embedded in the collagen matrix contracted less when treated with control nanoparticles at 100 nM siRNA compared to the fibroblasts treated with control siRNA at 50 nM (Fig. 7B). This decreased contractility of fibroblasts in collagen matrix could be explained by the toxic and potential off-target effects of the nanoparticles at 100 nM siRNA concentration. siRNAs can induce non-specific effects on protein levels that are not siRNA sequence dependent as siRNAs may cross-react with targets of limited sequence similarity^29^,^30^. However, siRNA off-target effects can be significantly reduced when fibroblasts are treated with a dose of siRNAs that is relatively low but sufficient to effectively silence the intended gene target^31^.

## Discussion

Fibrosis remains a critical determinant of the long-term surgical success after glaucoma filtration surgery and small molecule therapeutics hold a lot of potential to modulate post-surgical wound healing in the eye^32^. In this study, we have developed a receptor-targeted liposome-peptide-siRNA nanoparticle incorporating MRTF-B siRNA. The nanocomplexes self-assemble at optimal ratios of cationic liposomes, targeting peptides and siRNAs, with the peptides providing siRNA packaging and cell targeting functions^5^,^24^ while the lipids help to modulate the surface properties of the nanoparticles, such as PEG-mediated hydrophilicity^6^, and assist the endosomal escape within the cell after endocytosis of the nanocomplexes^33^,^34^. The MRTF-B siRNA-loaded nanoparticles were successfully taken up by human Tenon’s fibroblasts to deliver the therapeutic siRNAs into the fibroblasts. Over 70% *MRTF-B* gene silencing was achieved after a single transfection treatment, demonstrating that efficient gene silencing can be achieved by this approach. Interestingly, this level of gene silencing by the liposome-peptide-siRNA nanoparticles in human Tenon’s fibroblasts was also effective to completely block matrix contraction for the whole duration of the 7-day functional contraction assay.

Nanotechnology and nanoparticles currently represent an area of great research interest due to the translational potential in a wide variety of scientific fields. Our next aim is to validate our results *in vivo* by testing the different nanoparticle formulations in the rabbit model of glaucoma filtration surgery. The rabbit model of glaucoma filtration surgery represents a very aggressive scarring response compared to that in humans^35^,^36^. It is a well-established model of ocular fibrosis and agents that have reduced scarring in the rabbit have been shown to be effective in humans in clinical trials^37^,^38^,^39^. Butler *et al.* have reported that topical silver nanoparticles led to a sustained reduction in intraocular pressure and blebs with decreased fibrosis and ischaemia in the rabbit model of glaucoma filtration surgery^40^. Furthermore, Ye *et al.* have tested subconjunctival injections of nano-copolymers [CS-g-(PEI-b-mPEG)/ IKKβ-siRNA] in a non-human primate model of glaucoma filtration surgery and found a significant improvement in the bleb survival and subconjunctival scarring compared to controls^41^.

Size and charge play a key role on the cellular uptake and cytotoxicity of nanoparticles. Several authors have shown an inverse relationship between size and cytotoxicity^42^,^43^,^44^,^45^,^46^ or cellular uptake^42^,^47^,^48^,^49^,^50^,^51^ of nanoparticles. Bhattacharjee *et al.* have reported that positive smaller polymer nanoparticles (45 nm) showed higher cellular uptake but higher cytotoxicity than positive bigger nanoparticles (90 nm)^42^. Possible mechanisms for the higher toxicity might be a reduction in mitochondrial membrane potential, uncoupling of the electron transfer chain in mitochondria and resulting ATP production, induction of reactive oxygen species and oxidative stress^42^. Zhang *et al.* have also performed a molecular modeling and thermodynamics study and have predicted higher cellular uptake for nanoparticles of about 44 nm in size^51^, due to stronger interactions with the receptors in receptor-mediated endocytosis. In this study, we have used positive receptor-targeted liposome-peptide-siRNA nanoparticles of about 100 nm and they have shown high silencing efficiency with the use of targeting peptides as well as low cytotoxicity.

Receptor-targeted delivery systems, using targeting peptides and ligands, have the key advantage of facilitating uptake into target cells and of preventing non-specific delivery into normal tissues^52^. In our study, receptor-targeted liposome-peptide-siRNA nanoparticles efficiently silenced the *MRTF-B* gene by 76% and 72% in human Tenon’s fibroblasts using the targeting peptides Y and ME27, respectively. Both peptides cyclise rapidly after dissolving by oxidation as we have previously described for a similar peptide^53^. ME27 contains a tripeptide Arg-Gly-Asp (RGD) motif that targets integrins, particularly α_v_β_3_, α_v_β_5,_ and α_5_β_1_, and these surface integrin receptors are abundantly expressed on human eye fibroblasts^54^. The peptide ligand YGLPHKF in peptide Y has been identified by biopanning a phage peptide library. Peptide Y closely resembles part of a targeting protein expressed by the intracellular pathogen Legionella pneumophila^5^,^22^ but the identity of the receptor is still unknown^55^. However, we have shown that peptide Y mediates the targeted delivery of siRNA in nanocomplexes to cells of neuronal origin^5^,^24^, lung cells^6^,^26^, primary vascular cells and rabbit aorta^22^,^23^, and is thus a peptide that could be used for different target tissues.

In addition, our study shows that non-PEGylated liposome formulations have higher silencing efficiency than PEGylated liposome formulations in human Tenon’s fibroblasts. Stealth coatings of nanocomplexes, for example by PEGylation, can enhance serum stability and minimise non-specific interactions, but can also affect cellular uptake and thus decrease the transfection efficiency of nanocomplexes^56^,^57^. The PEGylated nanoparticles in this study were slightly bigger in size than the non-PEGylated ones and also less cationic due possibly to PEG shielding, and these biophysical differences in conjunction with the lower cellular uptake might have contributed to the observed reduced silencing effect. Another potential reason could be that the peptide was not able to reach its cell target due to PEG shielding. However, since the PEGylation was performed in exactly the same way during the liposomal preparation as in our previously published work which showed high targeting specificity for anionic nanocomplexes with the same PEG moiety^6^,^25^, we did not anticipate that this would play a crucial role.

Toxicity is another major hurdle to overcome when developing new anti-fibrotic treatments in the eye. Lipofectamine is a very efficient transfection reagent but is however cytotoxic and cannot be used *in vivo* and in the eye^58^,^59^. The liposome-peptide-siRNA nanoparticles were not cytotoxic in human Tenon’s fibroblasts at 50 nM siRNA concentration, supporting their safety as a non-viral siRNA delivery system to the conjunctiva. Higher siRNA concentrations, e.g. 100 nM, however showed a decrease in cell viability compared to the untreated cells. In addition, nanoparticles using different targeting peptides (Y, ME27) and different liposomes (non-PEGylated, PEGylated) did not significantly affect cell viability compared to control nanoparticles and untreated cells. Ladewig *et al.* have shown that layered double hydroxide nanoparticles facilitated uptake of siRNAs into mammalian cells due to their small size (100 nm) and positive charge, and also showed low cytotoxicity^60^. However, large particle sizes and higher concentrations have been associated with increased cytotoxicity and genotoxicity^61^. Tan *et al.* did not observe any toxic side effects with the layer-by-layer nanoparticles^62^, and Ye *et al.* have also found that the cationic nano-copolymers were well tolerated in the eye^41^.

In conclusion, we have developed a receptor-targeted liposome-peptide-siRNA nanoparticle incorporating MRTF-B siRNA, targeting peptides, and cationic liposomes. The MRTF-B siRNA-loaded nanoparticles efficiently silenced the expression of the *MRTF-B* gene in human Tenon’s fibroblasts, were not cytotoxic, and completely blocked collagen matrix contraction after a single transfection treatment. Receptor-targeted liposome-peptide-siRNA nanoparticles thus represent an efficient and safe non-viral siRNA delivery system that could be used to prevent conjunctival fibrosis after glaucoma filtration surgery and other fibroblast-induced contractile scarring conditions in the eye.

## Methods

### Cell Culture

Primary human Tenon’s fibroblasts (HTFs) were isolated from donor eyes in the eye bank and informed consent was obtained from all subjects. All experimental protocols were approved by the institutional approval committee at the University College London Institute of Ophthalmology, and all the methods were carried out in accordance with the approved guidelines. Cells were maintained in Dulbecco’s modified Eagle’s medium (DMEM, Invitrogen) with 10% fetal calf serum (FCS), 100 U/ml penicillin, 100 mg/ml streptomycin, and 2 nM L-glutamine, in tissue culture incubators with 5% CO_2_ and 95% humidity. Cells between passages 2–8 were used in the experiments.

### Preparation of Nanoparticle formulations

The liposome-peptide-siRNA nanoparticle formulations were prepared at a weight ratio of 1 (liposome): 4 (peptide): 1 (siRNA), by first mixing the liposome (0.2 mg/ml in water) with the peptide (0.2 mg/ml in OptiMEM [Life Technologies, UK]), followed by the addition of siRNA (5 μM stock diluted in OptiMEM). The mixture was incubated at room temperature for one hour to allow complex formation, and then additional OptiMEM was included to give a final siRNA concentration of 50 nM or 100 nM. Different targeting peptides (Y, ME27) and different liposomes (non-PEGylated, PEGylated) were used to prepare the nanoparticle formulations. The structures of the different lipids and peptides are shown in Table 1. The non-PEGylated liposomes were made up of DOTMA/DOPE (1:1 molar ratio), and the PEGylated liposomes were made up of 47.5% DOTMA: 47.5% DOPE: 5% DPPE-PEG2000 in molar ratios. All the lipids were bought from Avanti polar lipids (Alabama, USA) and all the peptides were synthesised by ChinaPeptides (Shanghai, China). The nanoparticle formulations were also compared to lipofectamine reagent [Life Technologies, UK] using 50 nM siRNA and according to the manufacturer’s instructions.

HTFs were seeded at 1 × 10^5^ cells/well in 6-well plates (Falcon, Fisher Scientific UK) and incubated for 24 hours before the nanocomplexes were added. HTFs were incubated with the nanoparticle complexes in OptiMEM for 4 hours at 37 °C. Following the 4 hour incubation, the medium containing the nanoparticles was replaced by fresh growth medium and the cells were incubated for a further 48 hours at 37 °C. HTFs were then used for RNA extraction to measure the silencing efficiency by real-time qPCR, or lysed to detect the silencing at protein level by western blotting.

### Nanoparticle size, zeta potential measurements, morphology, and internalisation of siRNA complexes

Nanoparticle size and zeta potential were determined by dynamic light scattering and by laser Doppler anemometry respectively, using a Nano ZS Zetasizer (Malvern Instruments, Malvern, UK) with the following specifications: automatic sampling time of 10 measurements/ sample, refractive index of 1.330, dielectric constant 78.5, viscosity 0.8872 cP, and temperature of 25 °C. Zeta potential settings were calibrated against the standard (−68 mV ± 6.8 mV). Triplicate measurements were performed for each sample and the results were analysed using the software provided by the manufacturer (DTS version 5.03). The nanoparticles were also visualised using negative staining transmission electron microscopy (TEM) to study their morphology as previously described^26^.

1.5 × 10^5^ cells were seeded onto poly-L-lysine coated slides (SLS, Dublin, Ireland). The following day they were transfected with Cy3-labelled GAPDH siRNA (final concentration of siRNA was 200 nM; Applied Biosystems, Warrington, UK) complexed with our formulations made as described above in triplicates. After 4 hours of incubation, the slides were washed with phosphate-buffered saline (PBS) and fixed in 4% formaldehyde, permeabilised with 0.5% Triton, and stained for 45 minutes with AlexaFluor 488 phalloidin (1:200, Invitrogen, Paisley, UK). The slides were then washed and sealed in mounting media containing DAPI (Invitrogen, Paisley, UK), and imaged at a magnification of x400 on a Carl Zeiss LSM710 laser scanning microscope system (Jena, Germany) as previously described^24^.

### Small interfering RNA Transfection

*MRTF-B* was knocked down using a SMARTpool of siGENOME human MRTF-B siRNAs (Dharmacon), with the following target sequences: GAAAAGAGCUCGACUAGCA, GAACGAGCCAGAACUGAAA, GGAUGGAACUUUACCCUCA, UCAGAAGGGUGAGAAGAAU. HTFs were transfected with 50 nM or 100 nM of MRTF-B siRNAs or control siRNAs (AllStars Negative Control siRNAs, Qiagen).

### MTT Cell Assay

Cell viability was measured using the Vybrant® MTT Assay (Life Technologies, UK). HTFs were seeded in 96-well plates and transfected with liposome-peptide-siRNA nanoparticles as described above. After 48 hours, the nanoparticle complexes were replaced by fresh growth medium. 10 μl of the 12 mM MTT solution [3-(4,5-dimethylthiazol-2-yl)-2,5-diphenyltetrazolium bromide] were added to each well and incubated for 4 hours at 37 °C. After 4 hours, 50 μl of DMSO were added to each well and incubated for a further 10 minutes at 37 °C. Each experiment was carried out with six independent replicates for each condition. Absorbance was measured at 540 nm on a SpectraMax Plus 384 spectrophotometer (Molecular Devices, California, USA).

### Real-Time Quantitative PCR

HTFs were lysed for RNA extraction using the Sigma RNA isolation kit (Sigma-Aldrich, Dorset, UK) according to the manufacturer’s instructions. Reverse transcription was carried out using the Transcriptor First Strand cDNA Synthesis Kit (Roche) according to the manufacturer’s instructions. RT-qPCR reactions were performed using SYBR green reagents (Life Technologies) on an HT7900 Fast Real-Time PCR system (Applied Biosystems, Life Technologies). The primer sequences for MRTF-B and GAPDH are listed in Table 2. All mRNA values were normalised relative to that of GAPDH and a standard curve using human genomic DNA was used to quantify the mRNA levels for each condition. Each experiment was carried out as independent triplicates for each group.

### Western Blotting

Proteins were extracted from HTFs using 2 × SDS sample buffer (100 mM Tris HCL pH 6.8, 4% SDS, 20% glycerol, 200 mM dithiothreitol and 0.2% bromophenol blue). Equal amounts of protein were loaded onto and run on 4–12% NuPAGE Bis-Tris protein gels (Novex, Life Technologies). The gels were transferred onto nitrocellulose blotting membranes (Amersham, Life Sciences), and blocked in 3% non-fat milk in PBST (PBS 0.1% Tween) for 60 minutes. The membranes were then incubated overnight at 4 °C in primary antibody (MRTF-B, C-19 sc-47282, 1:1000, Santa Cruz; GAPDH, G9545, 1:3000, Sigma). The next day, the membranes were washed three times for 10 minutes each in PBST, and incubated for 1 hour at room temperature in secondary-labelled antibody (MRTF-B: Anti-goat HRP immunoglobulins, 1:2000, Dako; GAPDH: Anti-rabbit HRP immunoglobulins, 1:5000, Dako). The membranes were then washed with PBST three times for 10 minutes each, treated with ECL solution (Amersham, Life sciences) for 5 minutes, and scanned on an Odyssey IR Imager (LI-COR). MRTF-B protein silencing was also measured using densitometric analysis and GAPDH as loading control.

### Collagen Contraction Assays

HTFs were trypsinised and a cell suspension containing 1 × 10^5^ cells/ml was centrifuged at 1500 rpm for 5 minutes. The supernatant was aspirated and the cell pellet re-suspended in 100 μl of fetal calf serum. A collagen gel solution was prepared using 1 ml of Type I collagen (2.05 mg/ml in 0.6% acetic acid, First Link) and 160 μl of concentrated medium (1.4 mls DMEM [Sigma Aldrich], 140 μl L-glutamine [Life Technologies], 360 μl sodium bicarbonate 7.5% [Sigma Aldrich]). The collagen solution was rapidly adjusted to pH 7 with sodium hydroxide and the cells were then added to the collagen solution. The fibroblast-populated collagen gel mixture was quickly cast into the wells of Mat Tek dishes (MatTek Corp, MI, USA), and left to set in the incubator for 10 minutes. Each gel was then detached from the edges of the well and put back into the incubator with 2 ml of growth medium. Whole matrix contraction was measured using digital images taken immediately following release of the polymerised matrices (t_0_) and then daily for 7 days (t_n_). The images were imported into Image J software (http://rsb.info.nih.gov/ij/). Gel surface area was normalised to the area calculated at t_0_ using the following formula: A (t_n_) in % = 100 − (100 × r_tn_^2^/r_to_^2^), where A is the gel surface area and r is the radius. Each experiment was performed as triplicates of matrices for each condition.

### Statistical Analysis

All graphs display mean and standard deviation (SD). Statistical analysis was performed using the Student’s t-test to calculate statistically significant differences and individual *P* values. Statistically significant differences were expressed as *P < 0.05, **P < 0.01.

## Additional Information

**How to cite this article**: Yu-Wai-Man, C. *et al.* Receptor-targeted liposome-peptide-siRNA nanoparticles represent an efficient delivery system for MRTF silencing in conjunctival fibrosis. *Sci. Rep.* **6**, 21881; doi: 10.1038/srep21881 (2016).

## Figures and Tables

**Table 1:**
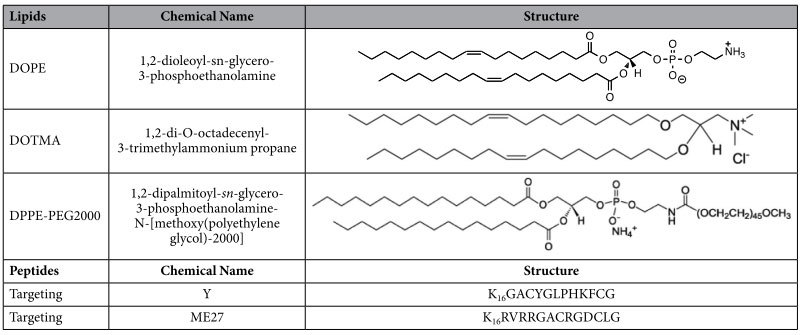
Structures of the different lipids and peptides.

**Table 2:** List of primers and their sequences.

Primers		Sequences
*MRTF-B*	F	CTTCCTGTGGACTCCAGTG
	R	TGTGACTCCTGACTCGCAG
*GAPDH*	F	GAAATGTGCTTTGGGGAGGC
	R	GGGGACAGGACCATATTGAGG
